# (In)congruence effect of chronological age and subjective age on older adults’ cognitive functioning: the moderating role of social participation and cross-cultural comparisons

**DOI:** 10.1186/s12877-026-07848-2

**Published:** 2026-06-30

**Authors:** Jiahui Li, Qiao Zhang, Mengze Wang, Baoshan Zhang

**Affiliations:** School of Psychology, Shaanxi Normal University, 199 South Chang’an Road, Yanta District, Xi’an, 710062 China

**Keywords:** Subjective age, Cognitive functioning, Social participation, Response surface analysis, Cultural differences

## Abstract

**Background:**

Chronological age and subjective age independently predict cognitive functioning, but their nonlinear interplay and the role of social participation remain less understood, particularly across cultures. This study aimed to investigate the (in)congruence effect of chronological and subjective age on cognitive functioning in older adults, to examine the moderating role of social participation in these associations, and to explore potential cultural differences.

**Methods:**

Longitudinal data from China (*N* = 4,314) and the US (*N* = 2,097) were analyzed using polynomial regression and moderated response surface analysis.

**Results:**

In both samples, individuals with congruently older chronological–subjective age (i.e., both chronological and subjective age were high) exhibited poorer cognitive outcomes. Under incongruence, cognitive functioning varied systematically depending on the relative discrepancy between chronological and subjective age, such that better outcomes were observed when chronological age was lower than subjective age compared to the opposite condition. In the Chinese sample, relatively younger subjective age was associated with more favorable cognitive outcomes, particularly among individuals with higher chronological age, whereas this pattern was not observed in the US sample. Moreover, social participation in the Chinese sample attenuated the negative congruence effect associated with older chronological–subjective age. It further altered the incongruence effect, such that a localized advantage of relatively younger subjective age was observed under low social participation, whereas no clear advantage was observed under high social participation, which was characterized by more complex nonlinear patterns. In contrast, no moderating effect of social participation was found in the US sample.

**Conclusions:**

These findings provide novel evidence for the interactive effects of objective and subjective age, psychosocial resources, and cultural context on cognitive aging, highlighting the potential importance of age perceptions and social participation in cognitive aging.

**Supplementary Information:**

The online version contains supplementary material available at https://doi.org/10.1186/s12877-026-07848-2.

## Introduction

Cognitive functioning typically declines with advancing age, yet the rate and extent of this decline vary substantially across individuals [1]. Such variability suggests that cognitive aging cannot be understood solely by biological processes but reflects the interplay of biological, psychological, and social factors. The life-course perspective provides an integrative framework for capturing this complexity, emphasizing that aging reflects interactions between objective time (e.g., chronological age), subjective experiences of time (e.g., subjective age), and broader sociocultural contexts [2, 3]. Within this framework, both chronological age and subjective age represent key temporal dimensions of aging.

In particular, subjective age—the age individuals feel themselves to be—represents an important component of age identity, capturing how individuals perceive, interpret, and adapt to age-related expectations and experiences across the life course [1]. Accumulating evidence has shown that subjective age, as well as chronological-subjective age discrepancies, are associated with cognitive outcomes in older adults [4, 5]. However, most existing studies conceptualize these associations in linear or additive terms, focusing on the independent effects of chronological age, subjective age, or their simple difference. Such approaches overlook the possibility that congruence and incongruence between chronological and subjective age may have distinct and potentially nonlinear implications for cognitive aging, as implied by the life-course perspective. That is, cognitive outcomes may differ not only when individuals feel younger or older than their chronological age (the incongruence effect), but also when both chronological and subjective age are jointly older or younger (the congruence effect).

The life-course framework further highlights the role of linked lives and social pathways in individual development [2, 3]. One key manifestation of these pathways is social participation, which embodies engagement in meaningful roles and activities [6, 7]. By providing opportunities for cognitive stimulation and role continuity, social participation may mitigate risks or support resilience, thereby moderating the (in)congruence effect of chronological and subjective age on cognition. Moreover, aging is embedded within cultural contexts that shape norms, values, and behavioral pathways, implying that the (in)congruence effect of chronological and subjective age on cognition, as well as the moderating role of social participation, may vary across cultural contexts [2, 3].

Building on these considerations, the present study examines how chronological and subjective age jointly relate to later cognitive functioning in older adults, whether social participation moderates these associations, and whether these patterns differ across cultural contexts. Because congruence and incongruence between chronological and subjective age inherently represent a two-dimensional configuration, traditional linear approaches are insufficient. Therefore, this study employs polynomial regression and response surface analysis to model nonlinear joint effects without reducing age differences to a single discrepancy score, providing a rigorous test of life-course theoretical propositions [8].

### Effect of chronological age and subjective age on cognitive functioning

Chronological age is commonly used as a temporal indicator of cognitive aging, reflecting the accumulation of age-related biological and neurological changes. Across diverse cultural contexts, increasing age is associated with reduced performance in processing speed, working memory, and executive function [9–11]. Processing resource theory suggests that these declines reflect reduced cognitive resources that constrain efficient information processing [12, 13]. Neuroscientific evidence further reveals that structural and functional brain changes, such as cortical atrophy, white matter deterioration, and disrupted neural connectivity, underlie these cognitive losses [14, 15].

Beyond chronological age, subjective age has emerged as an important psychological correlate of cognitive functioning. Evidence from the Midlife in the United States (MIDUS) and the Health and Retirement Study (HRS) indicates that feeling younger is associated with better episodic memory and executive functioning [5, 16, 17]. Consistent with this evidence, findings from the Health and Retirement Study (HRS) and the National Health and Aging Study (NHATS) showed that an older subjective age predicted heightened risk of cognitive impairment and incident dementia [18, 19]. The association between subjective age and cognitive outcomes may be explained through psychosocial, behavioral, and health-related pathways. For instance, individuals who feel younger are more likely to resist negative aging stereotypes and maintain active, engaged lifestyles that protect cognitive health [17, 20]. Thus, subjective age may capture psychological resources relevant to cognitive functioning.

### (In)congruence effect of chronological age and subjective age on older adults’ cognitive functioning

While both chronological and subjective age are independently associated with cognition, their congruence or incongruence may provide additional insight into age-related adaptation [21]. Within the subjective aging framework, discrepancies between chronological and subjective age can be understood as indicators of alignment or misalignment between objective aging and individuals’ subjective age identity, thereby signaling different forms of psychological adaptation to aging [22, 23]. When chronological and subjective age are congruent, it may indicate a stable age identity. Congruence at a younger level (low chronological and subjective age) can yield a dual advantage: individuals benefit biologically from relatively limited age-related decline due to younger chronological age, while also deriving psychological benefits from feeling younger, such as greater motivation and reduced stereotype threat. Together, these advantages may promote optimal cognitive functioning. Conversely, congruence at an older level (older chronological and subjective age) may represent a compounded risk for cognitive decline [24, 25]. When chronological and subjective age are incongruent, feeling younger or older than one’s actual age, may differentially impact cognitive functioning. Feeling younger than one’s chronological age is often associated with more favorable cognitive outcomes [5, 26]. This protective effect may stem from younger subjective age mitigating stereotype threat and promoting engagement in cognitively stimulating activities [20, 27]. In contrast, feeling older is typically linked to poorer cognition, likely due to internalized age stereotypes and reduced motivation to sustain cognitive vitality [28]. Overall, these perspectives suggest that cognitive functioning is likely to vary across different configurations of chronological and subjective age rather than being determined by either dimension in isolation.

### The moderating role of social participation

Social participation, encompassing activities such as volunteering, leisure, and community engagement, is a key protective factor for cognitive health [6, 7]. According to cognitive reserve theory, engagement in cognitively and socially stimulating activities may help maintain cognitive functioning [29]. Beyond its direct protective role, social participation may moderate the association between chronological–subjective age (in)congruence and cognitive functioning. From a life-course perspective, chronological age and subjective age jointly reflect both objective constraints and subjective experiences of aging, and their (in)congruence may signal varying levels of vulnerability or resilience [2, 3]. A key implication of this perspective is that these differences in vulnerability or resilience reflect how individuals draw on available resources, such that the cognitive consequences of chronological–subjective age (in)congruence depend on how these resources are utilized [30, 31]. In this context, subjective age may be conceptualized as an internal psychological resource, whereas social participation represents a key external psychosocial resource. When external resources are more or less available, the extent to which internal resources contribute to cognitive functioning may change. Accordingly, social participation may interact with subjective age in shaping cognitive functioning, thereby moderating the association between chronological–subjective age (in)congruence and cognitive functioning.

These moderating processes may operate differently depending on whether chronological and subjective age are congruent or incongruent. Under conditions of congruence, chronological and subjective age are aligned, reflecting relatively stable configurations of aging experience. In such cases, the role of social participation may depend more on baseline levels of advantage or risk. This pattern is consistent with cumulative advantage and disadvantage theory [33], which posits that psychosocial resources, such as social participation, produce incremental gains under low-risk conditions but primarily serve a compensatory function under high-risk conditions by offsetting accumulated vulnerabilities. Accordingly, when both chronological and subjective age are relatively low (double advantage), social participation may amplify cognitive benefits by reinforcing an active lifestyle and providing additional cognitive and social stimulation [34, 35]. By contrast, when both chronological and subjective age are relatively high (double risk), social participation may buffer cognitive decline by compensating for accumulated biological and psychological risks through enhanced social integration and cognitive engagement [7, 27]. In contexts of incongruence, the relative contribution of subjective age to cognitive functioning may depend on the availability of external resources such as social participation. From the perspective of resource substitution theory [32], when social participation is low, individuals may rely more heavily on internal psychological resources, such as a younger subjective age, to maintain cognitive functioning. When social participation is high, however, the protective value of a younger subjective age may be attenuated, as greater external engagement reduces reliance on internal resources.

### Cultural differences

Cultural context may shape how the congruence or incongruence between chronological and subjective age influences cognitive functioning. In collectivist cultures such as China, age identity is closely intertwined with social roles and intergenerational expectations, and subjective age may reflect perceived alignment with these age-normative standards [37]. Chronological–subjective age discrepancies may reflect a departure from expected role timing and may be associated with role-related processes, with implications for cognitive functioning depending on the direction and context of the discrepancy. In contrast, in individualistic cultures such as the United States, subjective age may be more strongly shaped by personal ideals of youthfulness and individual autonomy [38]. In these contexts, discrepancies between chronological and subjective age may carry different meanings, reflecting self-evaluative orientations rather than alignment with socially prescribed roles. As a result, these discrepancies may be more closely linked to internal psychological processes, thereby shaping the strength and form of the association between chronological–subjective age (in)congruence and cognitive functioning [17, 26]. Overall, the association between chronological–subjective age incongruence and cognitive functioning may differ across cultural contexts, as variations in the psychological meaning of age discrepancies may shape the strength and form of their relationship with cognition.

In addition, the moderating role of social participation in the association between chronological–subjective age (in)congruence and cognitive functioning may vary across cultural contexts. In collectivist contexts such as China, social participation is often embedded in social roles and intergenerational relationships, which may reinforce the salience of age-related expectations and shape how chronological–subjective age discrepancies are integrated into individuals’ social roles, thereby influencing their association with cognitive functioning [39]. In contrast, in individualistic contexts such as the United States, social participation is more likely to be self-directed and oriented toward personal preferences and autonomy, which may allow individuals to selectively engage in activities aligned with their self-perceptions, thereby shaping how chronological–subjective age discrepancies are linked to cognitive functioning [36]. These contextual differences suggest that social participation may operate differently in moderating the association between chronological–subjective age (in)congruence and cognitive functioning. However, it remains unclear whether the moderating role of social participation reflects universal psychological processes or culturally embedded patterns of aging, underscoring the need for systematic cross-cultural investigations.

### Current study

A better understanding of the life-course perspective requires assessing nonlinear effects of chronological and subjective age on older adults’ cognitive functioning, as well as the social and cultural conditions under which these effects unfold. However, previous studies have paid limited attention to these aspects. This study aims to employ polynomial regression combined with response surface analysis on longitudinal data from the China Longitudinal Aging Social Survey (CLASS) and the Health and Retirement Study (HRS). Specifically, we investigate [1] the (in)congruence effect of chronological and subjective age on cognitive functioning [2], whether social participation moderates the (in)congruence effect of chronological and subjective age on cognitive functioning, and [3] whether these (in)congruence effects and the moderating role of social participation differ between China and the US.

## Method

### Participants and procedure

This study drew upon data from two nationally representative and longitudinal cohort studies—the China Longitudinal Aging Social Survey (CLASS) [40] and the Health and Retirement Study (HRS) conducted in the United States [41]. In the CLASS cohort, subjective age was first assessed in the third wave (2018); therefore, the present study utilized data from Wave 3 (2018) to Wave 4 (2020), with Wave 3 designated as the baseline (T1). Correspondingly, data from Wave 13 (2016) to Wave 14 (2018) of the HRS were selected to align temporally with the CLASS data, with Wave 13 serving as the baseline (T1). At both T1 and T2, chronological age, subjective age, cognitive functioning, social participation, self-rated health, and depressive symptoms were assessed. Ethical approval for the CLASS and HRS studies was obtained from their respective Institutional Review Boards. All participants provided written informed consent prior to participation in both studies.

To ensure data quality and comparability between cohorts, several exclusion criteria were applied. In the CLASS cohort, 9,161 participants were successfully matched across Waves 3 (2018) and 4 (2020). Of these, 3,643 participants (39.8%) were excluded because of missing data on one or more key study variables, resulting in 5,518 participants. In the HRS cohort, subjective age and social participation were assessed through the Leave-Behind Questionnaire (LBQ), which is administered to a rotating random subsample of respondents rather than the full HRS cohort. Consequently, 5,410 participants had available LBQ data and were successfully matched across Waves 13 (2016) and 14 (2018). Among these participants, 902 (16.7%) were excluded because of missing data on one or more key study variables, resulting in 4,508 participants.

Subsequently, to enhance comparability between the two samples, only participants aged 65–85 years were retained. Participants younger than 65 years were excluded because the present study focused on older adults, whereas participants older than 85 years were excluded because the oldest-old often represent a highly selective group characterized by distinct survival advantages and increased measurement variability, which may bias age-related trajectories [42, 43]. Accordingly, 1,183 participants were excluded from the CLASS sample and 2,389 participants were excluded from the HRS sample based on age criteria. Finally, participants whose chronological age or subjective age values exceeded ± 3 standard deviations from the sample mean were removed, following previous RSA research [8]. This resulted in the exclusion of 21 participants from the CLASS sample and 22 participants from the HRS sample. The final analytic samples consisted of 4,314 respondents from the CLASS and 2,097 respondents from the HRS. The participant selection process for both cohorts is presented in the Supplementary Material (Figure S1). Participant characteristics for the Chinese and US samples are reported in the Supplementary Material (Table S3).

### Measures

#### Chronological age

In both the CLASS and HRS cohorts, chronological age was computed based on the difference between each participant’s date of birth and the date of the interview.

#### Subjective age

In both the CLASS and HRS cohorts, subjective age was measured by a single item: “How old do you feel that you are?”

#### Cognitive functioning

Class assessed cognitive functioning via the modified Mini-Mental Status Examination (MMSE) [44], including time orientation (0–3 points), place orientation (0–2 points), immediate recall (0–3 points), delayed recall (0–3 points), and serial-7 subtraction (0–5 points). The scores ranged from 0 to 16, with higher scores indicating better cognitive performance.

In the HRS cohort, Cognitive functioning was measured via the modified Telephone Interview for Cognitive Status (TICS-m) [45]. The TICS-m is a 27-point global cognitive scale that includes immediate and delayed recall of a 10-word list (0–20 points), counting backward (0–2 points), and serial-7 subtraction (0–5 points). The scores ranged from 0 to 27, with a higher score indicating better cognitive functioning.

#### Social participation

In the CLASS cohort, social participation was measured by the frequency of engagement in 13 activities across four domains (volunteering, religious, leisure, and learning activities) during the past year. Each activity was rated on a 5-point scale ranging from 0 (Never) to 4 (Almost Daily). The resulting total score ranged from 0 to 52, with higher scores indicating more frequent social participation.

In the HRS cohort, social participation is assessed by 21 questions that capture the frequency of involvement in various activities, including caregiving, volunteering, club activities, leisure activities, playing games, and physical activities [46]. The questions are 7-point Likert scale based (1 = Daily, 2 = Not in the last month, 3 = At least once a month, 4 = Several times a month, 5 = Once a week, 6 = Several times a week, 7 = Never/Not Relevant), the total score ranged from 21 (lowest) to 147 (highest). Higher scores indicate lower levels of social participation.

#### Covariates

Covariates included gender, educational level, T1 self-rated health, T1 depressive symptoms, and T1 cognitive functioning. Self-rated health was assessed in both the CLASS and HRS using a single item: “Overall, how healthy do you feel?” Responses were recorded on a 5-point Likert scale (1 = Excellent, 5 = Poor). CLASS assessed depressive symptoms using the revised Center for Epidemiological Survey Depression Scale (CES-D) [47]. The scale consists of 9 items on a 3-point scale ranging from 1 (Not at all) to 3 (Often). Scale scores range from 9 to 27, with higher scores indicating higher levels of depression. Cronbach’s α at T1 and T2 were 0.62 and 0.67, respectively. HRS measured depressive symptoms via the eight-item version of the Center for Epidemiologic Studies Depression Scale (CES-D) [48]. Participants indicated (yes/no) whether they had experienced each symptom during the past week. The total score was calculated by summing six negative symptom items and two positively worded items (reverse-coded), yielding a range from 0 to 8. Cronbach’s alpha was 0.78 and 0.80 at T1 and T2, respectively.

### Data analytic strategy

Descriptive statistics were conducted using SPSS 26.0. In addition, baseline characteristics were compared between participants retained in the analytic sample and those excluded because of missingness. To examine (a) the effects of congruence and incongruence between chronological and subjective age at T1 on cognitive functioning at T2 across both cohorts, and (b) the potential moderating role of social participation at T1 on this (in)congruence effect, we employed polynomial regression and multilevel moderated second-order response surface analysis (RSA). All RSA models were conducted using the RSA 4.0 macro in SPSS 26.0 [49].

The RSA analytical procedure included four steps. First, all independent variables (chronological age and subjective age at T1) and the moderator (social participation at T1) were mean-centered to mitigate multicollinearity issues using the built-in centering function of the RSA 4.0 macro [50]. Second, a polynomial regression equation (Model 1) was estimated: Z = b_0_ + control variables + b_1_X + b_2_Y + b_3_  X^2^ + b_4_XY + b_5_Y^2^ + e, where Z is the dependent variable (T2 cognitive function), X (T1 chronological age) and Y (T1 subjective age) are two independent variables, b_0_ is the intercept, b_1_-b_5_ are the regression coefficients, and e is the error term. Additionally, we examined the R² and the change in R² (ΔR²) from Model (1) The ΔR² after adding the quadratic and interaction terms (X², XY, and Y²) evaluated their incremental predictive power. Third, a moderated polynomial regression equation (Model 2) was estimated: Z = b_0_ + control variables + b_1_X + b_2_Y + b_3_  X^2^ + b_4_XY + b_5_Y^2^ + b_6_W + b_7_WX + b_8_WY + b_9_WX^2^ + b_10_WXY + b_11_WY^2^ + e, where the meanings of Z, X, Y, b_0_, and e are the same as those in Equation (Model2). W (T1 social participation) is the moderating variable, and b_1_-b_11_ are the regression coefficients. We reported R² and ΔR² from model (2) ΔR² was evaluated to determine the incremental contribution of the polynomial terms and their interactions with the moderator ((X + Y+X^2^ + XY+Y^2^)*W). Finally, based on the regression coefficients (b_1_-b_5_), the RSA parameters (a_1_-a_4_) are obtained [51]. Specifically, for the line of congruence (LOC; X = Y), the slope (a_1_) and curvature (a_2_) reflect how the congruence between X and Y affect Z; for the line of incongruence (LOIC; X = -Y), the slope (a_3_) and curvature (a_4_) reflect how the incongruence between X and Y affect Z. The Z-hat value was used to compare the effects of different (in) congruence levels on the cognitive outcomes [52].

As a sensitivity analysis, the primary RSA models were re-estimated using full information maximum likelihood (FIML) in Mplus 8.8 to evaluate the robustness of the findings to missing-data handling procedures. For these analyses, the same age restrictions (65–85 years) and outlier exclusion criteria (± 3 SD) applied in the primary analyses were retained, but participants with missing values on study variables were not excluded. FIML uses all available information under the assumption that data are missing at random (MAR) and is widely recommended for handling missing data in longitudinal studies.

## Results

### Preliminary analysis

Baseline demographic, health-related, and study characteristics were compared between participants retained in the analytic sample and those excluded because of missingness (see Tables S1 and S2 for detailed descriptive statistics and group comparisons). Across both cohorts, excluded participants tended to be older, less educated, report poorer health, and exhibit lower levels of cognitive functioning than retained participants. Overall, these findings suggest that participants with greater age-related vulnerabilities were somewhat more likely to be excluded from the analytic sample.

The means, standard deviation, and correlations among key variables for both cohorts, are reported in the Supplementary Material (see Table S4). In the CLASS sample, 46.25% of participants showed congruence between T1 chronological age and T1 subjective age, while 53.75% showed incongruence. In the HRS sample, 40.34% were congruent and 59.64% were incongruent. These distributions satisfy the recommended criterion for polynomial regression, which requires a sufficient proportion (> 10%) of incongruent X and Y values to ensure reliable estimation [53].

### (In)congruence effect of T1 chronological age and T1 subjective age on T2 cognitive functioning

#### (In)congruence effects in the chinese sample (CLASS)

**Fig. 1:**
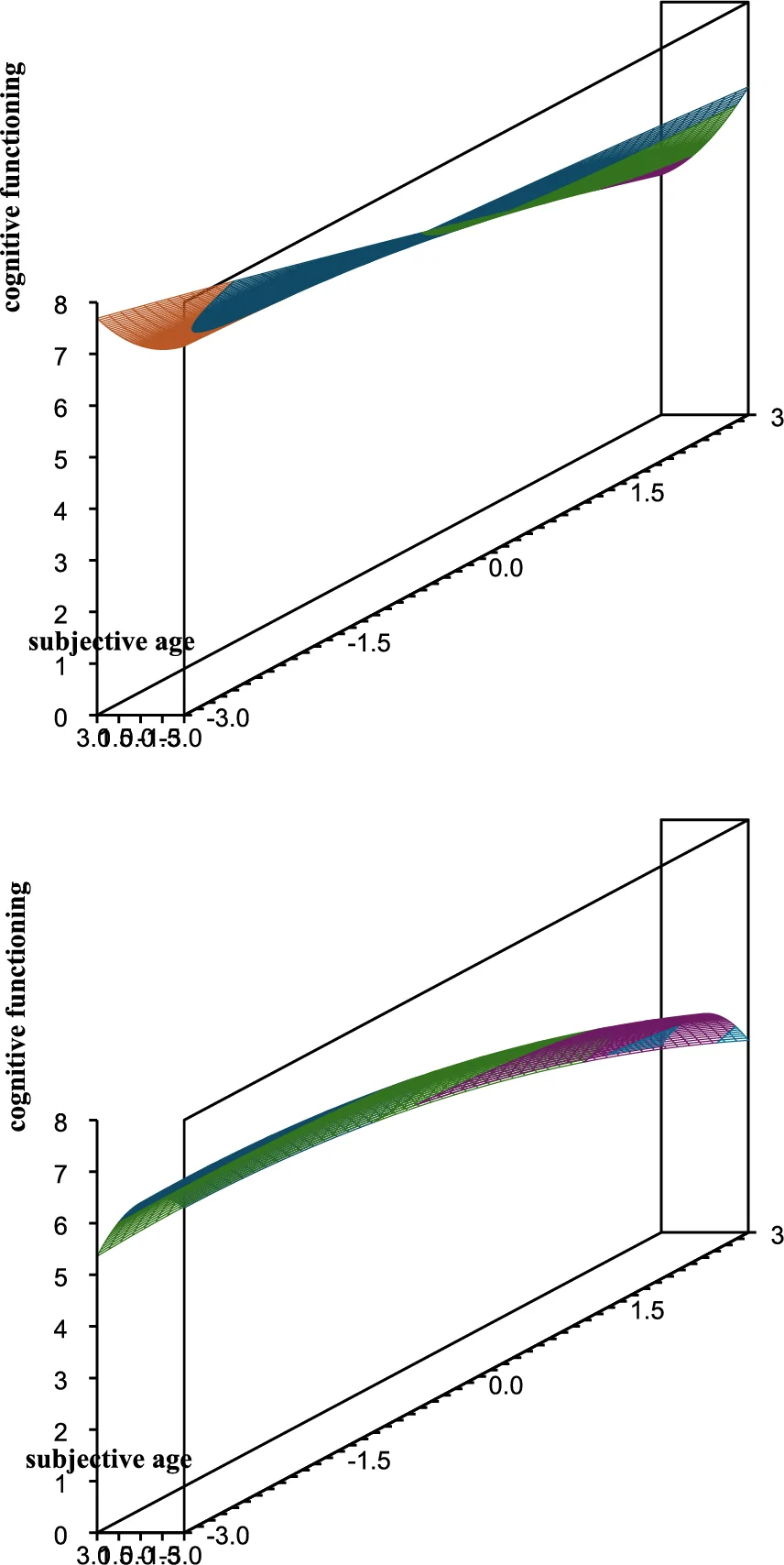
Response surfaces in the unconditional model: The (in)congruence effects of T1 chronological age and T1 subjective age on T2 cognition in China (**a**) and the US (**b**)

In CLASS model 1, after controlling for covariates, both T1 chronological age (*p* < 0.001) and T1 subjective age negatively predicted T2 cognitive functioning (*p* = 0.039). Moreover, the significant interaction between chronological and subjective age (*p* = 0.019) indicated interdependent nonlinear relationships between T1 chronological–subjective age and T2 cognition functioning (see Supplementary Material; Table S5). RSA parameters are presented in Table 1, and the response surface is illustrated in Fig. 1a. Analysis of the LOC revealed a significant negative slope (a_1_ = − 0.42, *p* < 0.001) but a non-significant curvature (a_2_ = − 0.04, *p* = 0.134), suggesting that when chronological and subjective age were aligned, cognitive functioning declined linearly as both ages increased (Z-hat = 0.94, *p* < 0.001). Along the LOIC, a significant slope (a_3_ = -0.22, *p* = 0.001) and nonsignificant curvature (a_4_ = 0.09, *p* = 0.105) demonstrated that the response surface followed a relatively linear decline from left to right. Specifically, compared to the condition in which T1 chronological age exceeded T1 subjective age, T2 cognition was significantly higher when T1 subjective age exceeded T1 chronological age (Z-hat = 0.45, *p* = 0.008). Notably, these comparisons reflect relative discrepancies between chronological and subjective age. This pattern reflects variation along the line of incongruence, indicating that cognitive outcomes differ depending on the direction of discrepancy between chronological and subjective age.

**Table 1:** Slopes and curvatures of LOC and LOIC in the unconditional model

	China: T2 cognition as the outcome		The US: T2 cognition as the outcome	
	B(SE)	95%CI	B(SE)	95%CI
a_1_ = b_1_ + b_2_	-0.42 (0.04)^⁎⁎⁎^	[-0.50, -0.33]	-0.45 (0.08)^⁎⁎⁎^	[-0.61, -0.28]
a_2_ = b_3_ + b_4_ + b_5_	-0.04 (0.03)	[-0.10, 0.01]	-0.11 (0.06)	[-0.23, 0.01]
a_3_ = b_1_–b_2_	-0.22 (0.07)^⁎⁎^	[-0.36, -0.09]	-0.27 (0.13)^⁎^	[-0.52, -0.02]
a_4_ = b_3_–b_4_ + b_5_	0.09 (0.05)	[-0.02, 0.19]	-0.16 (0.12)	[-0.39, 0.07]
a_5_ = b_3_–b_5_	-0.04 (0.03)	[-0.10, 0.03]	-0.01 (0.07)	[-0.15, 0.13]

The a_1_ coefficient represents the slope of the line of congruence (LOC); the a_2_ coefficient represents the curvature of the LOC; the a_3_ coefficient represents the slope of the line of incongruence (LOIC); the a_4_ coefficient represents the curvature of the LOIC; the a5 coefficient represents the position of the first principal axis. b_0_–b_5_ are coefficients in polynomial regression equation: Z = b_0_ + control variables + b_1_X + b_2_Y + b_3_ X ^2^ + b_4_XY + b_5_Y^2^ + e. The same thereafter; *p < 0.05, **p < 0.01, ***p < 0.001

In addition, this pattern varied across age levels. Among individuals with lower T1 chronological age, no significant differences in T2 cognitive functioning were observed between discrepancy conditions (Z-hat = 0.04, *p* = 0.802). However, among individuals with higher T1 chronological age, those in the chronological age > subjective age condition (i.e., relatively younger subjective age) exhibited significantly better cognitive outcomes than those in the chronological age < subjective age condition (Z-hat = 0.46, *p* = 0.004), suggesting that the discrepancy pattern differs across age levels, with a reversal in direction observed among individuals with higher chronological age.

#### (In)congruence effects in the US sample (HRS)

In HRS model 1, after controlling for covariates, T1 chronological age negatively predicted T2 cognitive functioning (*p* < 0.001), while T1 subjective age was not a significant predictor (*p* = 0.355). Among the polynomial terms, only the squared term of T1 chronological age was significant (*p* = 0.018), indicating a nonlinear association between T1 chronological age and T2 cognitive functioning (see Supplementary Material; Table S5). RSA parameters are shown in Table 1, with the corresponding response surface depicted in Fig. 1b. For the LOC, the slope was significantly negative (a_1_ = -0.45, *p* < 0.001), while the curvature was not significant (a_2_ = -0.11, *p* = 0.075), reflecting a near-linear downward trend from front to back. T2 cognitive functioning was significantly higher when both chronological and subjective age were younger compared to when both were older (Z-hat = 1.08, *p* < 0.001). Along the LOIC, the slope was also significantly negative (a_3_ = -0.27, *p* = 0.032), while the curvature was not (a_4_ = -0.16, *p* = 0.184), revealing that T2 cognitive functioning followed an approximately downward straight line from left to right. Compared to the condition in which T1 chronological age exceeded T1 subjective age, T2 cognitive functioning was significantly higher when T1 subjective age exceeded T1 chronological age (Z-hat = 0.72, *p* = 0.031). Furthermore, regardless of whether participants had younger or older chronological age at T1 (Z-hat = 0.26, *p* = 0.396; Z-hat = 0.10, *p* = 0.774), there were no significant differences in cognitive function between discrepancy conditions, indicating that the protective effect of relatively younger subjective age was not observed in the US sample.

### Moderating role of T1 social participation

#### Moderating role of social participation in the chinese sample (CLASS)

**Fig. 2:**
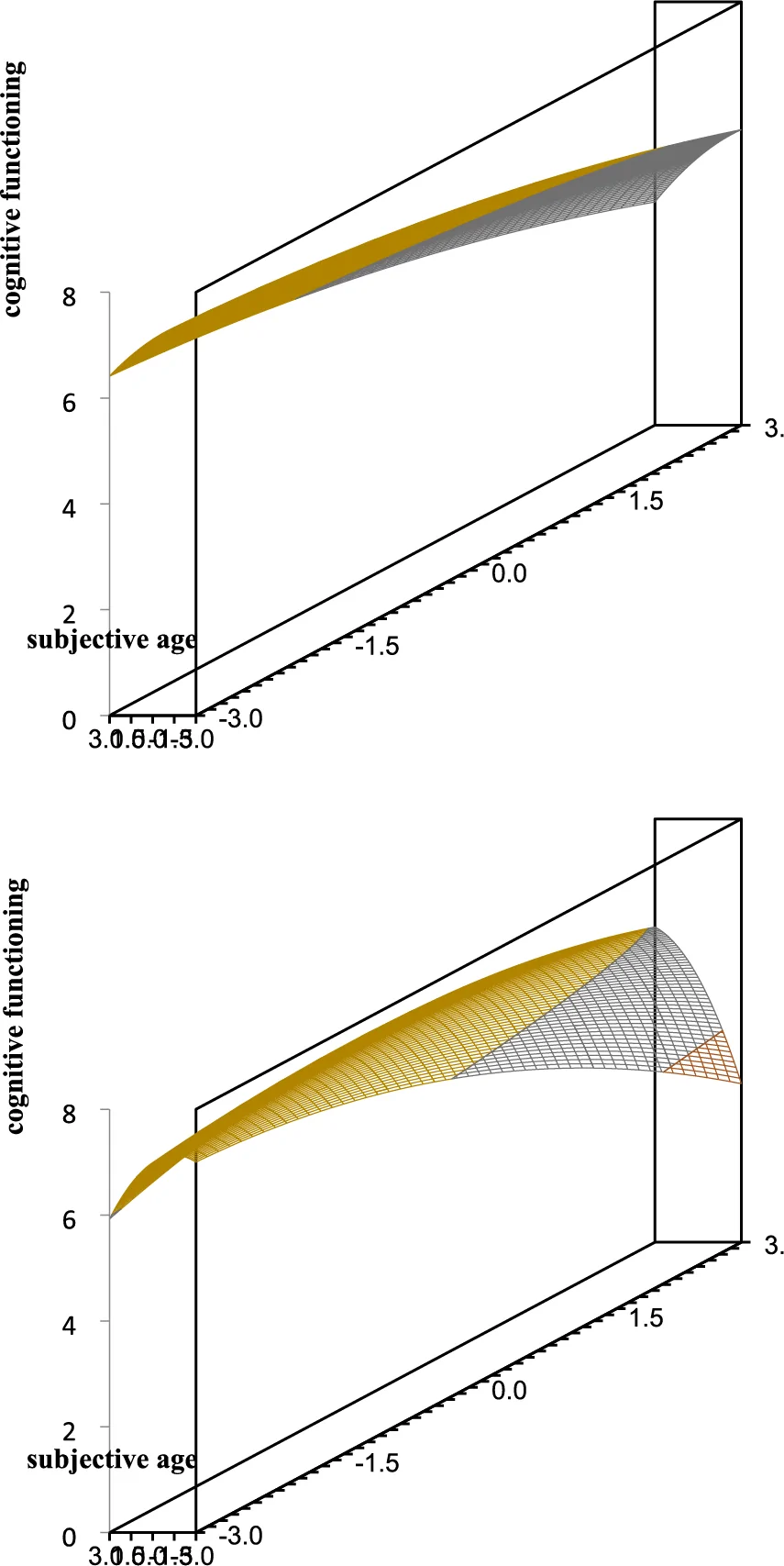
Response surfaces at low T1 social participation level: The (in)congruence effects of T1 chronological age and T1 subjective age on T2 cognition in China (**a**) and the US (**b**)

In CLASS Model 2, the inclusion of T1 social participation as a moderator significantly improved the model’s explanatory power (*p* = 0.003). Significant interaction effects were found for W×Y (*p* = 0.021) and W×X^2^ (*p* = 0.012), indicating a moderating role of T1 social participation (see Supplementary Material; Table S5). Results of the moderated RSA showed that under low levels of T1 social participation (*M*-1*SD*), the LOC had a significant negative slope (a_1_ = -0.54, *p* < 0.001) but a non-significant curvature (a_2_ = -0.07, *p* = 0.061), suggesting a near-linear decline in cognitive function as both chronological and subjective age increased (Z-hat = -1.17, *p* < 0.001). For the LOIC, both the slope (a_3_ = − 0.14, *p* = 0.133) and curvature (a4 = − 0.05, *p* = 0.480) were non-significant, indicating no significant variation along the line of incongruence. Accordingly, no significant differences in T2 cognition were observed between the T1 chronological age > T1 subjective age and T1chronological age < T1 subjective age conditions (Z-hat = 0.27, *p* = 0.244) (see Table 2; Fig. 2a). However, simple slope tests showed that, at both lower (Z-hat = 0.42, *p* = 0.032) and higher (Z-hat = 0.48, *p* = 0.027) levels of chronological age, individuals in the T1 chronological age > T1 subjective age condition exhibited significantly better cognitive functioning at T2. indicating an advantage of relatively younger subjective age under low levels of social participation.

**Table 2:** Slopes and curvatures of LOC and LOIC in models at different levels of T1 social participation

	China: T2 cognition as the outcome						The US: T2 cognition as the outcome			
	Low T1 SP (M-1SD)			High T1 SP (M+1SD)			Low T1 SP (M-1SD)		High T1 SP (M+1SD)	
	B(SE)	95%CI		B(SE)	95%CI		B(SE)	95%CI	B(SE)	95%CI
a_1_ = b_1_ + b_2_	-0.54 (0.06)^⁎⁎⁎^	[-0.65, -0.43]		-0.25 (0.07)^⁎⁎⁎^	[-0.40, -0.11]		-0.17(0.13)	[-0.43, 0.08]	-0.56(0.11)^⁎⁎⁎^	[-0.78, -0.35]
a_2_ = b_3_ + b_4_ + b_5_	-0.07 (0.04)	[-0.14, 0.00]		0.01 (0.04)	[-0.07, 0.10]		-0.04(0.10)	[-0.24, 0.15]	-0.06(0.08)	[-0.21, 0.10]
a_3_ = b_1_–b_2_	-0.14 (0.09)	[-0.32, 0.04]		-0.32 (0.11)^⁎⁎^	[-0.54, -0.10]		-0.49(0.20)^*^	[-0.87, -0.11]	-0.24(0.16)	[-0.56, 0.08]
a_4_ = b_3_–b_4_ + b_5_	-0.05 (0.07)	[-0.19, 0.09]		0.26 (0.09)^⁎⁎^	[0.07, 0.44]		-0.27(0.17)	[-0.59, 0.06]	-0.02(0.14)	[-0.30, 0.25]
a_5_ = b_3_–b_5_	-0.02 (0.04)	[-0.11, 0.06]		-0.05 (0.05)	[-0.15, 0.06]		-0.04(0.11)	[-0.25, 0.17]	-0.05(0.09)	[-0.22, 0.13]

SP = social participation. b_0_-b_10_ are coefficients in polynomial regression equation: Z = b_0_ + control variables + b_1_X + b_2_Y + b_3_ X ^2^ + b_4_XY + b_5_Y^2^ + b_6_W + b_7_WX + b_8_WY + b_9_WX^2^ + b_10_WXY + b_11_WY^2^ + e. *p < 0.05, **p < 0.01, ***p < 0.001

**Fig. 3:**
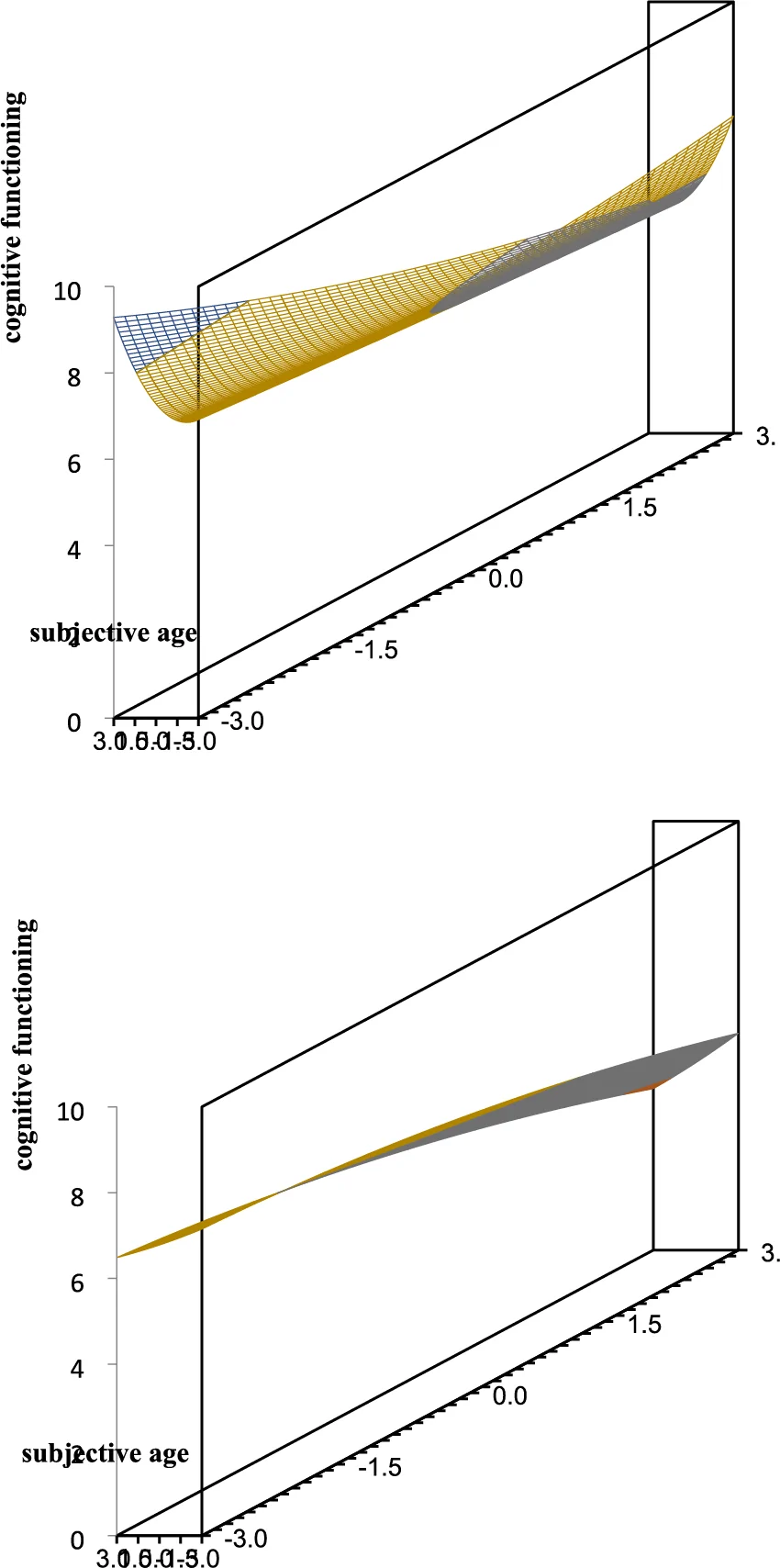
Response surfaces at high T1 social participation level: The (in)congruence effects of T1 chronological age and T1 subjective age on T2 cognition in China (**a**) and the US (**b**)

At high levels of social participation (*M*+1*SD*), the slope of LOC remained significant though attenuated (a_1_ = -0.25, *p* < 0.001), suggesting a weaker negative association between chronological–subjective age congruence and cognition. The curvature of LOC remained non-significant (a_2_ = 0.02, *p* = 0.717), indicating that T2 cognition followed an approximately straight line from front to back. Notably, LOIC showed a significant slope (a₃ = − 0.32, *p* = 0.004) and curvature (a₄ = 0.26, *p* = 0.006), forming a concave surface with the lowest cognitive function observed in the chronological age > subjective age region (stationary point: 30.70, 20.02) (see Table 2; Fig. 3a). For individuals with high levels of social engagement, no significant differences in T2 cognitive functioning were observed between the T1 chronological age > T1 subjective age and T1 chronological age < T1 subjective age conditions (Z-hat = − 0.38, *p* = 0.052 for lower chronological age; Z-hat = 0.43, *p* = 0.141 for higher chronological age), indicating no clear advantage of relatively younger subjective age.

#### Moderating role of social participation in the US sample (HRS)

In the HRS Model 2, including T1 social participation as a moderator did not significantly improve the explanatory power of the model (*p* = 0.132), and none of the interaction terms involving social participation reached significance (*p*s > 0.05). This indicates that T1 social participation did not moderate the (in)congruence effects of chronological and subjective age on T2 cognitive functioning in the US sample (see Supplementary Material; Table S5). Moderated RSA parameters are presented in Table 2, and the response surface is shown in Figs. 2b and 3b.

### Sensitivity analysis

To evaluate the robustness of the findings to missing-data handling procedures, the primary response surface models were re-estimated using full information maximum likelihood (FIML) estimation in Mplus after applying the same age restrictions (65–85 years) and outlier exclusion criteria (± 3 SD) used in the primary analyses. The resulting analytic samples consisted of 5,716 participants in the CLASS cohort and 2,436 participants in the HRS cohort. As shown in Table S6, the direction, magnitude, and statistical significance of the response surface parameters were highly similar to those obtained in the primary analyses. In particular, the significant congruence effect (a1) and incongruence effect (a3) remained statistically significant in both cohorts. These findings support the robustness of the primary conclusions to alternative missing-data handling procedures.

## Discussion

This study adopted a life-course perspective to examine how chronological age and subjective age, as objective and subjective dimensions of time, jointly relate to later cognitive functioning, and how these associations are shaped by social participation and broader contextual factors. Using longitudinal data from the CLASS and HRS cohorts and applying polynomial regression with moderated response surface analyses, we identified several key patterns. Across both samples, congruently older chronological–subjective age was associated with poorer subsequent cognitive outcomes. Under conditions of incongruence, cognitive functioning varied systematically depending on the relative discrepancy between chronological and subjective age, such that better outcomes were observed when chronological age was lower than subjective age. Notably, relatively younger subjective age was associated with more favorable cognitive outcomes in the Chinese sample, particularly among individuals with higher chronological age, whereas no such advantage was observed in the US sample. Finally, in the Chinese sample, social participation reduced the congruence effect by mitigating the cognitive disadvantage associated with older chronological–subjective age, while also altering the structure of the incongruence effect; conversely, no moderating effects were observed in the US sample. Together, these findings underscore the importance of psychosocial resources and cultural context in shaping how objective and subjective aging jointly influence cognitive trajectories.

### Effect of chronological age and subjective age on cognitive functioning

Consistent with extensive prior research, the present study found that chronological age was negatively associated with cognitive functioning in both China and the US. This pattern reflects the well-documented trajectory of age-related decline in memory, processing speed, and executive functioning [54]. Furthermore, within the US sample, a nonlinear association between chronological age and cognition was observed, suggesting that cognitive decline may accelerate at older ages, in line with prior work showing curvilinear age effects [55].

Beyond the role of chronological age, the findings highlight cross-national differences in the predictive effects of subjective age. In the Chinese sample, older adults who felt older relative to their chronological age exhibited poorer subsequent cognitive functioning, whereas feeling younger was associated with more favorable cognitive outcomes. This aligns with previous research linking younger subjective age to better cognitive outcomes and healthier aging trajectories [5]. In contrast, subjective age did not emerge as a significant predictor of cognitive performance in the US sample, despite the direction of associations aligning with prior research. This suggests that the predictive role of subjective age for cognition may differ across cultural contexts, as further elucidated in the subsequent analyses of (in)congruence effects.

### (In)Congruence effect of chronological age and subjective age on cognitive functioning

Regarding the congruence Effect, results across both samples revealed a linear decline in cognitive performance as chronological and subjective age simultaneously increased. This pattern suggests that when individuals are not only objectively older but also feel older, biological aging and psychological aging processes may converge to exacerbate cognitive decline. Such convergence may intensify negative self-perceptions of aging, activate maladaptive stereotypes, and reduce engagement in cognitively stimulating activities, thereby accelerating decline [55, 56].

Regarding the incongruence effect, a consistent pattern emerged across both samples, such that subsequent cognitive functioning was higher when chronological age was lower than subjective age (i.e., chronological age < subjective age) compared to the opposite condition. This pattern reflects a systematic variation along the line of incongruence, indicating that cognitive functioning differs depending on the relative discrepancy between chronological and subjective age, consistent with lifespan perspectives emphasizing the dynamic interplay between biological and psychological aspects of aging [57]. Notably, a more nuanced pattern was observed in the Chinese sample. Among individuals with higher chronological age, we found an age-dependent protective effect of younger subjective age (i.e., subjective age < chronological age), which was associated with better subsequent cognitive functioning. This suggests that the benefits of feeling relatively younger may become particularly relevant in later life, when biological aging processes accelerate and external demands on cognitive resources intensify [16]. This finding also supports the model of selection, optimization, and compensation (SOC), indicating that in advanced age, individuals increasingly rely on psychological and motivational resources to compensate for biological decline [58]. Therefore, a younger subjective age may reflect a growth-oriented and engaged mindset that supports cognitive resilience in later adulthood.

In contrast, the protective effect of feeling younger was not observed in the US sample. Importantly, this finding should not be interpreted as evidence that subjective age is unimportant in Western contexts. Rather, it suggests that the subjective age–cognition association may depend on contextual factors, including cultural norms, psychological processes, and measurement sensitivity. Consistent with this perspective, a recent meta-analysis reported stronger associations between subjective age and developmental outcomes in collectivist than individualist contexts [4]. One possible explanation is that in Eastern societies, age-related norms and intergenerational expectations may render age identity more salient in later life, thereby strengthening associations between subjective age and cognitive outcomes [59]. In contrast, in Western contexts, subjective age may be more closely tied to self-evaluative processes, which may be more strongly reflected in psychological experiences such as depressive symptoms [17, 26]. As a result, the association between subjective age and cognitive functioning may be less directly observable or may depend on intermediate processes that were not explicitly examined in the present study. However, these interpretations should be made with caution, as the present study did not directly assess cultural mechanisms, and the observed differences may also reflect broader country-level factors. In addition, measurement sensitivity may further contribute to these observed cross-national differences, as subjective age tends to show stronger associations with domain-specific cognitive outcomes (e.g., episodic memory and executive functioning) than with global indices [22, 60, 61]. Taken together, these findings should be framed as country-level differences that warrant further testing with harmonized, domain-specific measures.

### The moderating role of social participation

In the Chinese sample, social participation moderated the (in)congruence effects of chronological and subjective age on cognitive functioning. First, regarding the congruence effect, cognitive performance declined most steeply when both chronological and subjective age were older, reflecting a “dual risk” of objective and subjective aging. However, this decline was attenuated among individuals with higher levels of social participation, suggesting that social participation may serve as a crucial psychosocial resource in mitigating the vulnerabilities associated with older chronological–subjective age levels. According to activity theory, such engagement provides continuous cognitive stimulation, opportunities for information exchange, and fulfillment of social roles, which together function as “cognitive exercise” to counteract the decline associated with both advancing chronological age and subjective age [7, 62].

Second, social participation also moderated the incongruence effect of chronological and subjective age on cognitive functioning in the Chinese sample. Among highly engaged individuals, the response surface showed a significant slope (a3) and curvature (a4) along the line of incongruence, indicating a nonlinear pattern in which cognitive functioning varied systematically along the line of incongruence, with a general decline from the chronological age < subjective age region toward the opposite region. No clear advantage of relatively younger subjective age was observed under high levels of social participation. In contrast, among individuals with low social participation, no significant variation was observed along the line of incongruence. Simple slope tests further indicated that individuals in the chronological age > subjective age condition (i.e., relatively younger subjective age) demonstrated better cognitive functioning at both younger and older levels of chronological age. This pattern suggests a localized advantage of relatively younger subjective age under conditions of low social participation.

Taken together, these findings suggest that social participation may alter not only the strength but also the structural form of the association between chronological–subjective age incongruence and cognitive functioning. Under conditions of low social participation, the localized advantage of younger subjective age may reflect greater reliance on internal psychological resources. In contrast, under high social participation, no clear advantage of relatively younger subjective age was observed, and cognitive functioning appeared to be shaped by more complex, nonlinear interactions between chronological and subjective age [63]. This pattern is broadly in line with the compensatory model of resilience, which proposes that protective factors compensate for vulnerabilities when other resources are scarce [64]. Thus, the role of subjective age appears dynamic and context-dependent, varying with the availability of external resources.

In the US sample, social participation did not moderate the (in)congruence effects of chronological and subjective age on subsequent cognitive functioning. One possible interpretation is that the meanings and functions of social participation may differ across social contexts. Within the US context, older adults often engage in social activities primarily for personal enjoyment or self-fulfillment. While such activities can enhance well-being and life satisfaction, they may not always provide sufficient cognitive demands needed to support cognitive functioning in later life [65, 66]. Moreover, US older adults typically have access to diverse formal and informal resources (e.g., community services, structured programs, health-related support), which could diminish the unique role of social participation as a cognitive resource [67, 68]. This may explain why, unlike in the Chinese sample, higher social participation did not amplify or attenuate the (in)congruence effects. Overall, these findings suggest that the cognitive benefits of social participation are likely to be context-dependent, partly shaped by broader social and cultural factors.

### Limitations and implications

This study has several important limitations. First, although two-wave longitudinal data were used, the design does not allow strong inferences about within-person cognitive change or causal processes. The findings should therefore be interpreted as prospective associations rather than evidence of cognitive decline or protection over time. Future studies with additional waves are needed to more rigorously examine change trajectories and dynamic compensation processes. Second, a proportion of participants in both the CLASS and HRS datasets were excluded during data preparation, primarily due to missing values and predefined eligibility criteria. Such sample reduction is common in large-scale longitudinal studies of older adults [69], where incomplete responses and attrition are unavoidable. Comparisons between retained and excluded participants suggested that individuals with greater age-related vulnerabilities (e.g., older age, poorer health, and lower cognitive functioning) were somewhat more likely to be excluded from the analytic sample. Importantly, sensitivity analyses using full information maximum likelihood (FIML) yielded substantively similar results to those obtained in the primary analyses, supporting the robustness of the findings. Future studies would benefit from collecting more complete longitudinal data and minimizing participant attrition across waves. Third, cross-national comparisons are constrained by differences in measurement and survey design between the CLASS and HRS datasets. Although conceptually comparable constructs were used, differences in item content, scaling, and cultural framing may limit direct comparability. In addition, while some of the observed differences between the Chinese and US samples were discussed from a cultural perspective, the present study did not directly assess specific cultural mechanisms. Therefore, these findings should be interpreted as country-level differences that may also reflect other unmeasured contextual factors. Future cross-national research would benefit from harmonized measures, coordinated study designs, and explicit assessments of cultural constructs. Finally, cognitive functioning was assessed using a global index, which may obscure domain-specific associations with subjective age. Future research using domain-specific measures may help clarify these associations.

## Conclusion

This study shows that cognitive aging is shaped by the joint configuration of chronological and subjective age. The role of subjective age in cognitive functioning depends on its alignment with chronological age and is further shaped by social and cultural contexts. These findings advance the literature by highlighting a configurational perspective on subjective age and demonstrating its context-dependent role. By applying a response surface approach, this study provides a more nuanced characterization of age (in)congruence that captures nonlinear patterns not detectable using traditional methods. Overall, this study contributes to a more integrative understanding of cognitive aging by linking objective age, subjective age, and social participation within a contextualized framework, with implications for promoting cognitive health in later life.

## Supplementary Information

Supplementary Material 1.

## Data Availability

The data and analysis code used in this study are publicly available. Chinese data were obtained from the China Longitudinal Aging Social Survey (CLASS), and US data from the Health and Retirement Study (HRS). The complete analysis code and processed datasets are available on the Open Science Framework at https://osf.io/78ruj.

## Abbreviations

CLASS: The China Longitudinal Aging Social Survey
HRS: The Health and Retirement Study
MIDUS: The Midlife in the United States
NHATS: The National Health and Aging Study
TICS-m: The modified Telephone Interview for Cognitive Status
CES-D: The Center for Epidemiological Survey Depression Scale
