# Supplementary material for: (In)congruence effect of chronological age and subjective age on older adults’ cognitive functioning: the moderating role of social participation and cross-cultural comparisons
Source: BMC Geriatr. 2026 Jun 30;26:1190. doi: 10.1186/s12877-026-07848-2 (PMC13595529; doi:10.1186/s12877-026-07848-2)
Supplement: Supplementary file 1 [file 12877_2026_7848_MOESM1_ESM.docx]

**Table S1.** Comparison of participants retained and excluded due to missingness in the CLASS sample

| Variable | Excluded (*n*=3643) | Retained (*n*=5518) | *p* | Effect Size |
| --- | --- | --- | --- | --- |
| Age | 70.94 (6.67) | 70.03 (6.24) | < 0.001 | d = 0.14 |
| Female (%) | 0.492 | 0.501 | 0.366 | V = 0.01 |
| Education | 2.73 (1.35) | 3.01 (1.33) | < 0.001 | d = 0.21 |
| Self-rated health | 2.74 (0.96) | 2.61 (0.83) | < 0.001 | d = 0.14 |
| Depressive symptoms | 15.82 (3.04) | 15.62 (3.08) | 0.006 | d = 0.06 |
| Cognitive functioning | 12.91 (3.45) | 13.77 (2.78) | < 0.001 | d = 0.28 |
| Subjective age | 67.58 (8.65) | 66.94 (7.85) | 0.006 | d = 0.08 |
| Social participation | 4.75 (3.80) | 5.67 (4.60) | < 0.001 | d = 0.21 |

*Note***.** Effect sizes are reported as Cohen's *d* for continuous variables and Cramér's *V* for gender.

**Table S2.** Comparison of participants retained and excluded due to missingness in the HRS sample

| Variable | Excluded (*n* = 902) | Retained (*n* = 4508) | *p* | Effect Size |
| --- | --- | --- | --- | --- |
| Age | 68.67 (11.48) | 65.94 (10.77) | < 0.001 | d = 0.25 |
| Female (%) | 0.681 | 0.596 | < 0.001 | V = 0.07 |
| Education | 3.08 (1.37) | 3.57 (1.28) | < 0.001 | d = 0.38 |
| Self-rated health | 3.02 (1.03) | 2.79 (1.00) | < 0.001 | d = 0.23 |
| Depressive symptoms | 1.57 (1.98) | 1.31 (1.89) | < 0.001 | d = 0.14 |
| Cognitive functioning | 13.73 (4.48) | 15.71 (3.92) | < 0.001 | d = 0.49 |
| Subjective age | 58.90 (44.87) | 56.28 (17.65) | 0.006 | d = 0.10 |
| Social participation | 103.97 (16.97) | 99.13 (15.21) | 0.001 | d = 0.29 |

*Note***.** Effect sizes are reported as Cohen's *d* for continuous variables and Cramér's *V* for gender.

**Table S3.** Participant characteristics

| Variable | China | The US |
| --- | --- | --- |
|  | (*n* = 4314) | (*n* = 2097) |
| Sex, *n* (%) |  |  |
| Male | 2166 (50.2%) | 860 (41.0%) |
| Female | 2148 (49.8%) | 1237 (59.0%) |
| Age, *M* (*SD*) | 71.47 (5.21) | 73.79 (5.81) |
| Education, *n* (%) |  |  |
| Low | 3947 (91.5%) | 260 (12.4%) |
| Middle | 294 (6.8%) | 733 (35.0%) |
| High | 73 (1.7%) | 1104 (52.6%) |
| Self-rated health (baseline), *M* (*SD*) | 2.65 (0.82) | 3.29 (0.85) |
| Depression (baseline), *M* (*SD*) | 15.79 (3.04) | 2.81 (0.99) |

*Note.* Education was recoded into three levels to enhance cross-national comparability. In the Chinese sample (CLASS), low education included no schooling, literacy classes, elementary school, and middle school (codes 1–4); middle education included high school and vocational school (code 5); and high education included college diploma, bachelor's degree, and above (codes 6–7). In the US sample (HRS), low education included less than high school (code 1); middle education included GED or high school graduate (codes 2–3); and high education included some college or college and above (codes 4–5).

**Table S4.** The means, standard deviation, and correlations of key variables

| Country | Variables | *M ± SD* | 1 | 2 | 3 | 4 | 5 |
| --- | --- | --- | --- | --- | --- | --- | --- |
| China | 1. T1 CA | 71.47 ± 5.21 | 1.000 |  |  |  |  |
|  | 2. T1 SA | 68.04 ± 7.13 | 0.529^***^ | 1.000 |  |  |  |
|  | 3. T1 cognition | 13.62 ± 2.84 | –0.228^***^ | –0.198^***^ | 1.000 |  |  |
|  | 4. T2 cognition | 13.43 ± 2.96 | –0.298^***^ | –0.220^***^ | 0.545^***^ | 1.000 |  |
|  | 5. T1 SP | 5.45 ± 4.50 | –0.134^***^ | –0.079^***^ | 0.033^*^ | 0.002 | 1.000 |
| The US | 1. T1 CA | 73.79 ± 5.81 | 1.000 |  |  |  |  |
|  | 2. T1 SA | 62.78 ± 11.52 | 0.477^***^ | 1.000 |  |  |  |
|  | 3. T1 cognition | 15.21 ± 3.96 | –0.213^***^ | –0.144^***^ | 1.000 |  |  |
|  | 4. T2 cognition | 15.39 ± 4.29 | –0.265^***^ | –0.191^***^ | 0.598^***^ | 1.000 |  |
|  | 5. T1 SP | 99.68 ± 15.23 | 0.096^***^ | 0.211^***^ | –0.351^***^ | –0.342^***^ | 1.000 |

*Note.* CA = chronological age; SA = subjective age; SP = social participation. ^*^*p* < .05. ^**^*p* < .01. ^***^*p* < .001.

**Table S5.** Polynomial regression model examining the (in)congruence effects across different levels of social participation

|  | China: T2 cognition as the outcome | |  | The US: T2 cognition as the outcome | | |
| --- | --- | --- | --- | --- | --- | --- |
|  | Model1 | Model 2 |  | Model1 | Model 2 |  |
|  | B(SE) | B(SE) |  | B(SE) | B(SE) |  |
| Constant | 6.26 (0.34)^***^ | 6.24 (0.34)^⁎⁎⁎^ |  | 5.96 (0.50)^⁎⁎⁎^ | 6.42 (0.51)^⁎⁎⁎^ |  |
| Gender | -0.13 (0.08) | -0.10 (0.08) |  | 0.66 (0.15)^⁎⁎⁎^ | 0.54 (0.15)⁎⁎⁎ |  |
| Education | 0.14 (0.03)^⁎⁎⁎^ | 0.17 (0.03)^⁎⁎⁎^ |  | 0.42 (0.06)^⁎⁎⁎^ | 0.35 (0.07)⁎⁎⁎ |  |
| T1 SH | -0.21 (0.05)^⁎⁎⁎^ | -0.22 (0.05)^⁎⁎⁎^ |  | -0.31 (0.09)^⁎⁎⁎^ | -0.25 (0.09)⁎⁎ |  |
| T1 depression | 0.02 (0.01) | 0.02 (0.01) |  | -0.07 (0.05) | 0.51 (0.02)⁎⁎⁎ |  |
| T1 cognition | 0.50 (0.01)^⁎⁎⁎^ | 0.50 (0.01)^⁎⁎⁎^ |  | 0.53 (0.02)^⁎⁎⁎^ | -0.05 (0.05) |  |
| T1 CA (X) | -0.32 (0.03)^⁎⁎⁎^ | -0.31 (0.03)^⁎⁎⁎^ |  | -0.36 (0.05)^⁎⁎⁎^ | -0.37 (0.05)^⁎⁎⁎^ |  |
| T1 SA (Y) | -0.10 (0.05)^⁎^ | -0.08 (0.05) |  | -0.09 (0.09) | -0.003 (0.10) |  |
| T1 CA^2^ (X^2^) | -0.01 (0.02) | 0.001 (0.02) |  | -0.07 (0.03)^⁎^ | -0.07 (0.03)^⁎^ |  |
| T1 CA×SA (X×Y) | -0.06 (0.03)^⁎^ | -0.06 (0.03)^⁎^ |  | 0.02 (0.06) | 0.05 (0.06) |  |
| T1 SA^2^(Y^2^) | 0.03 (0.03) | 0.04 (0.03) |  | -0.06 (0.07) | -0.03 (0.07) |  |
| T1 SP (W) |  | -0.05 (0.01)^⁎⁎^ |  |  | -0.03 (0.01)^⁎⁎⁎^ |  |
| W×X |  | 0.01 (0.01) |  |  | -0.002 (0.004) |  |
| W×Y |  | 0.03 (0.01)^⁎^ |  |  | -0.01 (0.01) |  |
| W×X^2^ |  | 0.01 (0.004)^⁎^ |  |  | 0.002(0.002) |  |
| W×X×Y |  | -0.01 (0.01) |  |  | -0.004 (0.003) |  |
| W×Y^2^ |  | 0.01 (0.01) |  |  | 0.002 (0.004) |  |
| R^2^ | 0.34^***^ | 0.34^***^ |  | 0.41^***^ | 0.002^*^ |  |
| ΔR^2^ | 0.001^*^ | 0.003^**^ |  | 0.002^*^ | 0.002 |  |

*Note.* SH = self-rated health; CA = chronological age; SA = subjective age; SP = social participation. **p* < .05, ***p* < .01, ****p* < .001.

**Table S6.** Slopes and Curvatures of LOC and LOIC in the Unconditional Model Estimated Using Full Information Maximum Likelihood (FIML)

|  | China: T2 cognition as the outcome | | The US: T2 cognition as the outcome | |  |
| --- | --- | --- | --- | --- | --- |
|  | B(SE) | 95%CI | B(SE) | 95%CI |  |
| a1 = b1 + b2 | -0.40 (0.04)^***^ | [-0.50, -0.30] | -0.44 (0.08)^***^ | [-0.60, -0.29] |  |
| a2 = b3 + b4 + b5 | -0.04 (0.03) | [-0.09, 0.02] | -0.08 (0.06) | [-0.19, 0.04] |  |
| a3 = b1 − b2 | -0.22 (0.07)^**^ | [-0.36, -0.08] | -0.29 (0.12)^*^ | [-0.54, -0.05] |  |
| a4 = b3 − b4 + b5 | 0.06 (0.05) | [-0.03, 0.14] | -0.26 (0.14) | [-0.53, 0.00] |  |
| a5 = b3 − b5 | -0.02 (0.03) | [-0.08, 0.04] | -0.003 (0.08) | [-0.15, 0.14] |  |

*Note.* Results were estimated using full information maximum likelihood (FIML) after applying the same age restrictions (65–85 years) and outlier exclusion criteria (±3 SD) used in the primary analyses. The direction, magnitude, and statistical significance of the response surface parameters were highly similar to those obtained in the primary analyses. LOC = line of congruence; LOIC = line of incongruence. ^*^*p* < .05. ^**^*p* < .01. ^***^*p* < .001.

**2020 wave**

**(n=11,398)**

**r wave)**

**Participants successfully matched across waves (n=9,161)**

**2018 wave**

**(n=11,419)**

**Participants with complete data**

**(n=5,518)**

**Participants aged 65–85 years**

**(n=4,335)**

**Final analytic sample after excluding participants whose chronological age or subjective age exceeded ±3 SD from the sample mean(n = 4,314)**

**CLASS**

**2018 wave**

**(n=17,146)**

**r wave)**

**Participants with available LBQ measures**

**successfully matched across waves (n=5,410)**

**2016 wave**

**(n=20,912)**

**Participants with complete data**

**(n=4,508)**

**Participants aged 65–85 years**

**(n=2,119)**

**Final analytic sample after excluding participants whose chronological age or subjective age exceeded ±3 SD from the sample mean(n = 2,097)**

**HRS**

**Figure S1.** Data management process.
